# Animal or Plant: Which Is the Better Fog Water Collector?

**DOI:** 10.1371/journal.pone.0034603

**Published:** 2012-04-03

**Authors:** Thomas Nørgaard, Martin Ebner, Marie Dacke

**Affiliations:** 1 Biology Department, Lund University, Lund, Sweden; 2 Institute for Geosciences, University of Tübingen, Tübingen, Germany; University of Oxford, United Kingdom

## Abstract

Occasional fog is a critical water source utilised by plants and animals in the Namib Desert. Fog basking beetles (*Onymacris unguicularis*, Tenebrionidae) and Namib dune bushman grass (*Stipagrostris sabulicola*, Poaceae) collect water directly from the fog. While the beetles position themselves optimally for fog water collection on dune ridges, the grass occurs predominantly at the dune base where less fog water is available. Differences in the fog-water collecting abilities in animals and plants have never been addressed. Here we place beetles and grass side-by-side in a fog chamber and measure the amount of water they collect over time. Based on the accumulated amount of water over a two hour period, grass is the better fog collector. However, in contrast to the episodic cascading water run-off from the grass, the beetles obtain water in a steady flow from their elytra. This steady trickle from the beetles' elytra to their mouth could ensure that even short periods of fog basking – while exposed to predators – will yield water. Up to now there is no indication of specialised surface properties on the grass leafs, but the steady run-off from the beetles could point to specific property adaptations of their elytra surface.

## Introduction

**Figure 1:**
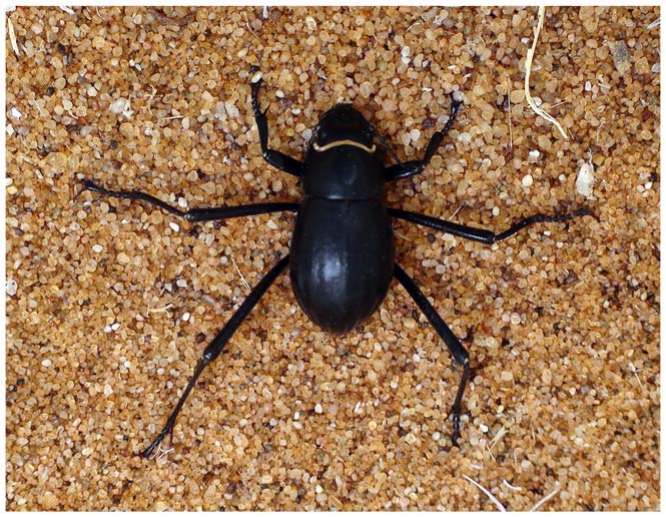
Fog basking beetle. The Namib Desert fog basking beetle *Onymacris unguicularis* (beetle length ca. 2 cm).

**Figure 2:**
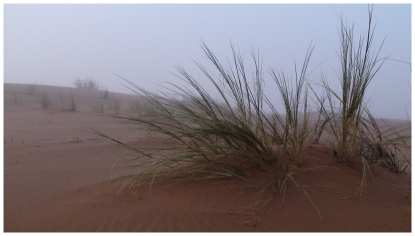
Namib dune bushman grass. A Namib dune bushman grass *Stipagrostris sabulicola* hummock in the fog. (plant height ca. 1 m).

The Namib Desert is one of the most arid habitats on Earth [1]. Rainfall is minimal and highly unpredictable, but fog from the Southern Atlantic can reach up to 100 Km inland and occur on 60–200 days per year [2]. During such fog events an average of one litre of water are deposited per square meter on artificial fog water collectors [3]. The fog is therefore a comparatively predictable source of water [4] and many life forms in the Namib Desert exhibit adaptations to utilize this. Most obtain water from the fog indirectly by drinking droplets deposited on external physical objects, while a few catch fog water directly from the air, using their own bodies as fog water collectors [5]–[8]. The fog basking beetle *Onymacris unguicularis* (Fig. 1) and the Namib dune bushman grass *Stipagrostris sabulicola* (Fig. 2) are examples of organisms collecting water directly from the air [5], [9]. During a fog event the beetles walk up to the top of a dune ridge and choose the optimal position with respect to wind direction for fog water collection [5]. Unstable substrate conditions and sand abrasion makes plant growth at the dune ridges difficult and *S. sabulicola* therefore predominantly settles at the dune base where substrate conditions are far more stable [10]. Fog density in arid regions increases with altitude [11] and due to local orographic effects [12], [13] less fog water precipitates at the dune base than up on the dune ridge [9]. The mobile beetles and immobile plants therefore have very different options and constraints when collecting fog water. Here we explore how this is reflected in their fog collecting abilities.

## Materials and Methods

Fog basking beetles and Namib dune bushman grass were collected in the Namib Desert (Beetles: 23°20′S, 14°47′E; Grass: 23°34′S, 15°03′E) and brought to Lund University in Sweden. All necessary permits were obtained for the described field studies. The collections of beetles and grass were done in collaboration with the National Museum of Namibia under permit issued to the museum by the Ministry of Environment and Tourism. In the laboratory the collected grass samples were kept fresh in a closed container at 5°C. The beetles were kept in sand-filled containers at 24°C, 12 h∶12 h light∶dark, and ca. 45% RH. All experiments were conducted within three weeks of collection. The fog collecting efficiency was tested by placing beetles killed by light freezing and 100 mm long sections of grass in a fog chamber. The fog chamber consisted of a 50 L refrigerator (Waves wc-16007) where temperature was kept between 10–15°C. This is comparable to the temperatures during a Namib Desert fog event [8]. Fog travelling ca. 0.1 m/s was generated with a fog producing machine (325 ml per hour) (Super fog, Lucky Reptile). Beetles were positioned at the 23° angle previously established as the mean angle between horizontal and ventral body surface of *O. unguicularis* in fog basking stance [14]. The grass was positioned at the same angle. Eppendorf tubes were placed to catch water running off the experimental objects and fog water harvesting efficiency was measured as the amount of water collected in the tubes. To test for specialised surface properties of the grass, sections of metal wire (galvanized iron) with similar dimensions (length = 100 mm and diameter = 1.4 mm) were included in the experiments. Like the beetles and grass straws, the metal wires were also positioned at a 23° angle. In the first set of experiments, the three experimental objects (beetle, grass straw, and metal wire) were placed in a row in random order and exposed to fog for two hours. Twelve experiments were performed and each individual experimental object used only once.

The size of the surface area exposed to fog will affect the amount of water collected. To calculate water collection per mm^2^ we determined the upper surface area of the experimental objects. The upper surface area A_u_ of the grass straw and metal wire sections was calculated as: A_u_ = π×Ø×L/2, where Ø and L is object diameter and length (L = 100 mm). The diameter of the individual grass straw sections was measured with calipers. The upper surface area of the irregularly shaped beetles was determined by coating them with coloured latex and then photographing the latex casts pressed flat under a glass plate. A photo of a one cm^2^ coloured square was used as a reference and the number of coloured pixels converted into a measure of mm^2^. The differences in the fog collecting efficiency between the three experimental object types were tested using ANOVA statistics and Tukey-Kramer Multiple Comparisons Post Hoc Test. The data were log transformed to pass Bartlett's test and Gaussian distribution was tested using the Kolmogorov–Smirnoff test. The results of the fog-water harvesting and the experimental object size calculations are all stated as mean values ± standard deviation.

In a second series of experiments, a set of twelve beetles were placed in the fog chamber and the amount of water collected after 0 to 120 minutes was measured in 20 minute increments. The six different experiment durations were presented in random order. The procedure was repeated with a set of twelve grass straws. The water run-off dynamics of the beetles and grass were compared using linear regression. An unpaired t-test with Welch correction was applied to test for difference in amount of fog water collected after two hours.

## Results

During two hours in the fog chamber the beetles collected 60.51±15.14 µl of water. This was significantly less (p<0.01) than the 111.94±44.53 µl, and 134.89±44.65 µl collected by the grass straws and metal wires, respectively. No significant difference was found between the two latter experimental object groups (p>0.05). The beetles' upper surface area was 245.27±34.59 mm^2^, the grass straws' 252.64±31.34 mm^2^, and the metal wires' was 219.91 mm^2^ (There was no measurable variation in metal wire diameter).

**Figure 3:**
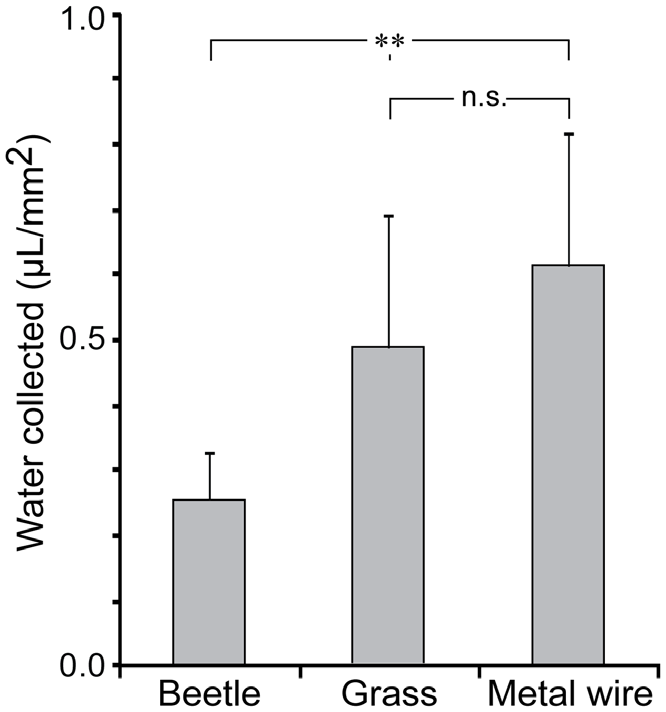
Fog water collection. Fog water collection (mean plus standard deviation) per mm^2^ of the experimental objects upper surface area. The fog basking beetles were found to harvest significantly less fog water than the grass straws and metal wires. One-way ANOVA, significance codes: ** p<0.01; n.s., not significant.

The amount of fog water collected per mm^2^ was calculated from the total upper surface area of the experimental objects. The beetles were found to collect 0.25±0.08 µl/mm^2^. This was again significantly less (p<0.01) than the 0.48±0.20 µl/mm^2^, and 0.61±0.20 µl/mm^2^ collected by the grass straws and metal wires, respectively (Fig. 3). No significant difference was found between the amount of fog water collected by the grass straws and metal wires (p>0.05).

**Figure 4:**
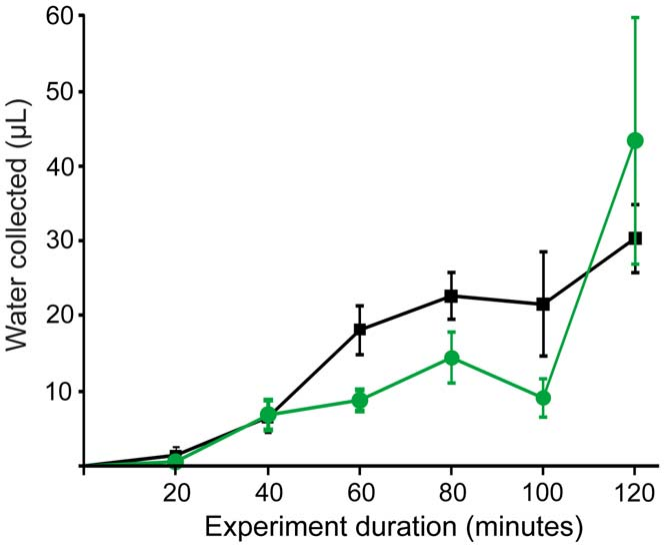
Water run-off dynamics of beetle and grass. Amount of water collected by beetles (black line, N = 12) and grass (green line, N = 12) exposed to fog in intervals from zero to 120 minutes with 20 minute increments (mean ± SD). The water run-off in the beetles is a steady flow, in plants it is in cascades.

The water run-off dynamics of beetles and grass proved to be very different (Fig. 4). After 100 minutes in the fog chamber the grass had only deposited 8.98±2.57 µl fog water in the sampling tubes. Just 20 minutes later this amount of collected water had increased almost fivefold to 43.45±16.37 µl. This episodic cascading water run-off dynamics results in a linear regression r^2^ = 0.54 (p<0.05). In contrast, the fog water deposited on the beetles' elytra run-off at a steady rate that results in a linear regression r^2^ = 0.85 (p<0.05). After 120 minutes the absolute amount of fog water collected by the grass was significantly higher than that collected by the beetles (P<0.001).

## Discussion

In this study we find that fog basking beetles are significantly less efficient at collecting water from fog laden air than the Namib dune bushman grass. This difference was present both in the absolute amount of water collected over a two hour period, as well as in the amount of water collected per mm^2^ of fog collecting area. Interestingly, the metal wire pieces collected the same amount of water as the grass straws. This suggests that the three dimensional structure of the grass straw – rather than any particular surface properties – is the important factor for its water collecting abilities. The slender shape of a Namib dune bushman grass leaf – or a metal wire – will probably produce a smaller boundary layer than the bulkier shape of the beetle [15] and this could enhance the water collecting ability of the grass over that of the beetle.

Differences between the water collecting abilities of the beetles and the grass were identified also in the temporal domain. Whereas the beetles showed a steady water run-off throughout the entire 120 minute period in the fog chamber, the grass leafs showed a strong increase in the amount of deposited water after 100 minutes. Up until this point, the beetles had in fact collected more water (Fig. 4). The steady water run-off from the elytra at a constant rate suggests that the beetles have elytra surface adaptations facilitating a frequent, if not constant, supply of water to the fog basking beetle. The beetle –exposed at the ridge of the dune – can thus afford to escape into the protective sand at any moment if disturbed.

Beetles in their natural habitat may have active behavioural ways of improving fog water harvesting. Had it been possible to use live beetles in this study, the fog collecting dynamics of the beetle could possibly have looked somewhat different. By carefully positioning the recently killed specimens head down in a close imitation of the characteristic fog basking posture of live beetles [14], we mimicked the natural situation as closely as possible.

In this comparative study between organisms as different as a plant and an insect, we show that the fog-basking beetles are less efficient at collecting fog water than the bushman grass. The challenges a desert plant and an animal face, and the constraints they operate under to meet these challenges, are very different. The beetle can reach the ridges of the dune, but needs a fog harvesting elytra that withstands abrasion from sand grains when diving into the loose sand. The beetle operating under the threat of desert predators might be interrupted at any moment in time and would therefore benefit from a steady water collecting rate. The Namib dune bushman grass occurs predominantly at the dune base where substrate conditions are stable, but much less fog water is deposited. The grass thus needs to be an efficient water harvester, which is also what we observe. The observed differences in fog collecting ability of the beetle and the grass raises questions about the mechanisms causing steady versus stochastic water collecting rates. Future experiments will elucidate the differences in surface structure between the two organisms and also address the importance of a steady water-collecting rate for the survival of the beetles in their natural surroundings. However, if the aim is to make biomimetic structures for fog water collection, we suggest it to be a better choice to focus on the grass rather than on the beetle.
